# Inverse Correlation of Cholesterol Efflux Capacity with Peripheral Plaque Volume Measured by 3D Ultrasound

**DOI:** 10.3390/biomedicines11071918

**Published:** 2023-07-06

**Authors:** Maria Noflatscher, Monika Hunjadi, Michael Schreinlechner, Philip Sommer, Daniela Lener, Markus Theurl, Rudolf Kirchmair, Axel Bauer, Andreas Ritsch, Peter Marschang

**Affiliations:** 1 Department of Internal Medicine III (Cardiology, Angiology), Medical University of Innsbruck, Anichstr. 35, A-6020 Innsbruck, Austriaphilip.sommer@student.i-med.ac.at (P.S.);; 2 Department of Internal Medicine I, Medical University of Innsbruck, Anichstr. 35, A-6020 Innsbruck, Austria; 3 Department of Internal Medicine, Central Hospital of Bolzano (SABES-ASDAA), Via Lorenz Boehler 5, I-39100 Bolzano, Italy

**Keywords:** atherosclerosis, 3D ultrasonography, cholesterol efflux capacity

## Abstract

Introduction: Cardiovascular disease (CVD) is a systemic multifocal illness called atherosclerosis that causes artery constriction and blockage. By causing cholesterol to build up in the artery wall, hypercholesterolemia is a major factor in the pathophysiology of atherosclerotic plaque development. Reverse cholesterol transport is the process of transporting cholesterol from the periphery back to the liver through cholesterol efflux mediated by high-density lipoprotein (HDL). It was suggested that the cholesterol efflux capacity (CEC), which is inversely linked with cardiovascular risk, can serve as a stand-in measure for reverse cholesterol transport. In this work, we sought to investigate a potential link between the peripheral plaque volume (PV) and CEC. Methods: Since lipid-lowering therapy interferes with CEC, we performed a cross-sectional study of 176 patients (48.9% females) with one cardiovascular risk factor or known CVD that did not currently take lipid-lowering medication. CEC was determined using cAMP-treated ^3^H-cholesterol-labeled J774 cells. Cholesterol ester transfer protein (CETP)-mediated cholesterol ester transfer was measured by quantifying the transfer of cholesterol ester from radiolabeled exogenous HDL cholesterol to Apolipoprotein B-containing lipoproteins. PV in the carotid and the femoral artery, defined as the total PV, was measured using a 3D ultrasound system equipped with semi-automatic software. Results: In our patients, we discovered an inverse relationship between high total PV and CEC (*p* = 0.027). However, there was no connection between total PV and low-density lipoprotein cholesterol, lipoprotein (a), or CETP-mediated cholesterol ester transfer. Conclusion: In patients not receiving lipid-lowering treatment, CEC inversely correlates with peripheral atherosclerosis, supporting its role in the pathophysiology of atherosclerosis.

## 1. Introduction

Atherosclerosis is a systemic multifocal disease leading to cardiovascular disease (CVD) [1]. Hypercholesterolemia plays a pivotal role in the pathogenesis of atherosclerotic plaques via the accumulation of cholesterol in the intimal layer of the arterial wall. In high-earning nations, CVD is the most frequent cause of death [2]. Low plasma concentrations of high-density lipoprotein cholesterol (HDL-C) constitute an important independent risk factor for the development of atherosclerosis and CVD [3]. However, therapeutically, attempts to increase HDL-C concentrations failed to reduce the risk of cardiovascular events [4,5,6,7]. Therefore, new therapies focused on HDL-C functionality rather than HDL-C plasma values [8,9].

The primary function of HDL is to remove excess cholesterol from peripheral cells, such as cholesterol-loaded macrophages in the arterial wall, and to transport it back to the liver to be metabolized into bile salts and then excreted. This process is called HDL-mediated reverse cholesterol transport [10]. The initial rate-limiting step is the removal of free cholesterol from the macrophage plasma membrane. This process can be experimentally measured as the cholesterol efflux capacity (CEC) of human plasma and is inversely correlated with cardiovascular risk [11,12,13,14,15,16]. Moreover, a low CEC is a relevant biomarker for cardiovascular events and constitutes a risk factor for cardiovascular mortality [11,17,18]. Reverse cholesterol transport also involves the redirection of cholesterol esters to triglyceride-rich lipoproteins via the cholesterol ester transfer protein (CETP). CETP is a lipid transport protein responsible for exchanging triglycerides and cholesteryl esters (CE) between HDL and triglyceride-rich lipoproteins [19]. Although CETP is generally considered atherogenic, therapeutic trials with CETP inhibitors have proven unsuccessful so far [20]. In a previous study of 1620 statin-treated patients with pre-existing coronary artery disease (CAD), we observed significantly more cardiovascular events in patients with low plasma CETP concentrations compared to those with high plasma CETP concentrations [21]. CETP-mediated CE transfer and CEC provided scientific methods for gaining further insight into HDL metabolism and its functionality [12].

One study showed that reconstituted HDL given intravenously restored endothelial function in the brachial artery in patients with type 2 diabetes mellitus [22]. In vivo mice studies showed that intravenous administration of reconstituted HDL increased myocardial perfusion in wild-type mice but not in mice deficient in endothelial NO synthase [23]. Another function of HDL appeared to be the attenuation of platelet reactivity in vitro, which may contribute to the beneficial effect on cardiovascular risk [22].

A three-dimensional (3D) ultrasound is a promising approach for the non-invasive quantification of peripheral plaque volume (PV) [24] with broad availability and without radiation or contrast medium [25]. In a study of 5800 asymptomatic individuals, the measurement of peripheral PV was similarly effective at predicting major adverse cardiac events as coronary artery calcium [26]. Using a 3D ultrasound, we previously showed that carotid and femoral PVs increase with cardiovascular risk factors, the involvement of vascular beds, chronic kidney disease (CKD), and male sex [27,28]. In this paper, we analyzed the association of HDL-C function with peripheral atherosclerotic PV in patients not receiving lipid-lowering medication.

## 2. Methods

### 2.1. Study Population

**Figure 1:**
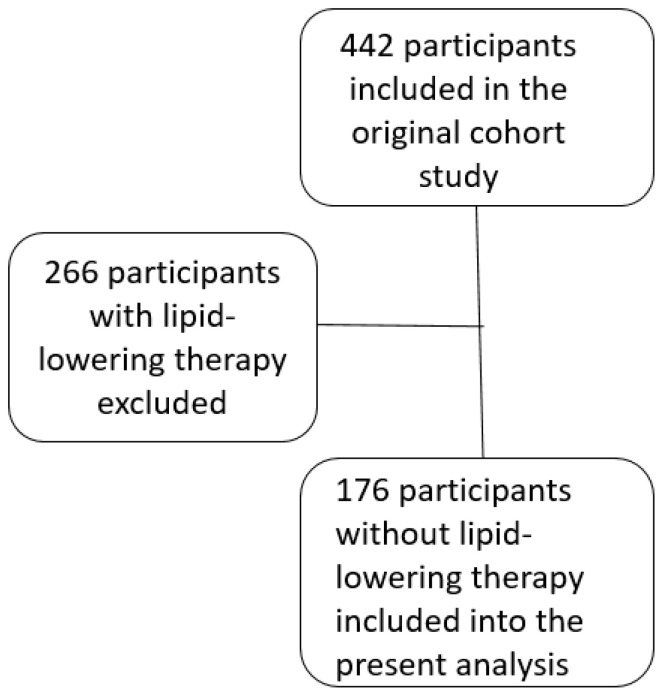
Flowchart of study participants.

The study “Correlation of Atherosclerotic PV and Intima-Media Thickness with Soluble P-selectin” (ClinicalTrials.gov Identifier: NCT01895725) was a prospective, observational single-center cohort study. The baseline criteria and inclusion criteria were published in detail in previous studies [27]. Briefly, patients with at least one cardiovascular risk factor or diagnosed CVD were eligible. Since lipid-lowering therapy interferes with CEC [29,30], we performed a cross-sectional analysis of a subgroup of this cohort at baseline, which comprised 176 subjects without current lipid-lowering therapy (see Figure 1). The characteristics of the 266 excluded participants and the present sample of 176 patients are presented in Table S1 (online supplement).

Each patient underwent a sonographic examination with quantification of the PV at baseline. An EDTA blood sample was concomitantly drawn from peripheral venous blood in addition to routine laboratory measurements. The EDTA blood sample was centrifuged at 1730× *g*, and the plasma was stored at −80 °C. Apolipoprotein (apo)B-depleted plasma was prepared by precipitating apoB-containing lipoproteins using 2.04 µL of 4% tungstophosphoric acid hydrate and 1.56 µL of MgCl_2_ to 25 µL of EDTA plasma, respectively.

This study’s plan was approved by the Ethics Committee of the Medical University of Innsbruck and complied with the Declaration of Helsinki. All study participants signed a written informed consent before enrollment into the study.

### 2.2. Ultrasound Imaging

The ultrasound determinations were already explained in detail in previous studies [27,28]. The Mannheim consensus was used to define the plaque volume [31] in the carotid and femoral arteries. The total PV was the summary of carotid and femoral PV calculated by Philips plaque quantification software (QLAB).

### 2.3. Cholesterol Efflux Capacity and Cholesterol Ester Transfer

CEC was quantified in plasma samples as described [1]. Briefly, 2.5 Ci/mL [^3^H] cholesterol was radiolabeled in J774.1A murine macrophage cells (ATCC#TIB-67), and ATP-binding cassette protein A1 was activated with 0.2 mM cAMP. The cells were then treated for four hours with efflux media containing 2.8% apoB-depleted plasma. At a concentration of 5 g/mL, an acyl-coenzyme A cholesterol acyltransferase inhibitor was used during the whole procedure. All plasma samples were taken in triplicate and kept at 80 °C before being measured. To measure the outflow of radioactive cholesterol from the cells, liquid scintillation counting was performed. The passive diffusion of [^3^H] cholesterol was measured using a plasma-free sample, which was employed as a background (negative control). To enable for the correction of discrepancies across tests throughout the plates, each plate had three control samples. According to the following equation, relative CEC was calculated as the proportion of control CEC in (%CEC): %CEC = ((DPM in media containing apoB-depleted plasma − mean DPM in acceptor-free medium)/(DPM in control sample − mean DPM in acceptor-free medium)) × 100.

CETP-mediated CE transfer was measured as previously described [29,30]. Briefly, CE transfer from exogenous HDL-C to apoB-containing lipoproteins was measured by incubating the whole plasma with radiolabeled [^3^H]-CE incorporated into HDL-C (less than 7% of sample HDL-C) and sodium iodoacetate as lecithin–cholesterol acyltransferase-inhibitor (Sigma, St. Louis, MO, USA) for 3 and 16 h, reflecting the onset rate depending more on the activity of the transfer-mediating enzyme CETP, and the total transfer rate depending on the concentration of the triglyceride-rich lipoproteins, respectively. ApoB-containing lipoproteins were isolated following dextran sulfate–magnesium chloride precipitation. CE transfer was measured as the radiolabeled CE rate for apoB-containing lipoproteins compared to controls containing a lipoprotein-depleted plasma sample. Results are expressed as the percentage decrease in [^3^H]-CE in the supernatant of total radiolabeled CE [32,33].

### 2.4. Statistical Analysis

Continuous variables that conform to a normal distribution tested with the Kolmogorov–Smirnov test [21] are reported as mean ± standard deviation (SD), while parameters that are not normally distributed are reported as median and interquartile range (IQR) or mean and 95% confidence interval. Bootstrapping was used to determine the 95% confidence intervals.

The categorical variables are given in absolute numbers and percentages. To compute differences between continuous variables and a categorical independent variable with two or more groups, the Mann–Whitney U test was applied. The chi-square test was used to compare categorical variables. Total PV was divided into high (501–2048 mm^3^) and low (0–500 mm^3^) total PV, with the 75th percentile as the cut-off point for some calculations, as described previously [34].

Receiver operating characteristic analysis was calculated to specify the predictive value of CEC for high total PV, the area under the curve (AUC), and the optimal cut-off value with the highest sensitivity and specificity. AUC values were compared using a nonparametric approach. The correlation between peripheral PV as a dependent variable and biomarkers, cardiovascular risk factors, and vascular disease as predictor variables was investigated using binary logistic regression. Only variables with *p* < 0.05 in univariate analysis were considered for multivariate regression. An intra-class correlation coefficient determined inter-observer variability for PV quantification. A *p*-value of <0.05 was considered significant. SPSS Statistic was used for all statistical analyses (version 24.0; IBM Corp., Armonk, NY, USA).

## 3. Results

### 3.1. Study Population

In this study, we included 176 patients (48.9% females) with a median age of 64 (IQR 57–70) without lipid-lowering therapy. All study participants received a 3D sonography of the carotid and femoral vessels. As mentioned already in previous studies [27,28], the inter-observer variability of three different observers revealed a high agreement of PV between the examiners, with an intra-class correlation coefficient of 0.95 (95% CI, 0.82–0.99). In addition, blood samples for routine laboratory examinations and an EDTA plasma sample for the determination of CEC and CE transfer were obtained. In Table 1, the baseline data are summarized.

About 15% of the study population suffered from CVD and CKD. Three-quarters of the participants had hyperlipidemia, and slightly more than half suffered from hypertension. About 20% of the study population had a family history of CVD, and 8.5% suffered from diabetes mellitus.

According to the plaque burden, the study population was divided into patients with low (<75th percentile) and high total PV (>75th percentile).

Participants with high total PV were significantly older and less likely to be female compared to those with low total PV. In addition, patients with high total PV were more likely to suffer from hypertension, CKD, and CVD, but less often they were found to have hyperlipidemia or high HDL-C levels. No significant differences between the two groups were observed for all other parameters (Table 1). The characteristics of the study participants (176 patients not receiving lipid lowering therapy) and the excluded 266 patients receiving lipid-lowering therapy of the original cohort are shown in Table S1 (online supplement). Apart from the expected differences in the lipid profile, the study participants were more likely to be female, had a lower BMI, smoked less frequently, and suffered less often from hypertension, diabetes, and vascular disease compared to the excluded patients.

### 3.2. Associations between CEC and High Total PV

**Figure 2:**
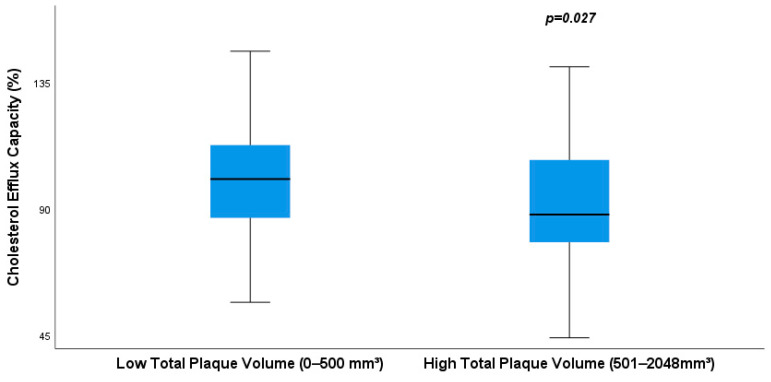
Distribution of cholesterol efflux capacity according to total plaque volume. Box plot represents the median and interquartile range of the cholesterol efflux capacity depending on the atherosclerotic plaque volume in patients without lipid-lowering therapy. The whiskers are representative of the 95% of the confidence interval.

We found that CEC values were inversely associated with high total PV (87.79% for high PV vs. 100. 41% for low PV, *p* = 0.027, Figure 2).

There was no significant correlation of high PV with the biomarkers CE transfer 3 h, CE transfer 16 h, lipoprotein (a), and low-density lipoprotein cholesterol (LDL-C).

### 3.3. Utility of CEC for the Prediction of High PV

**Figure 3:**
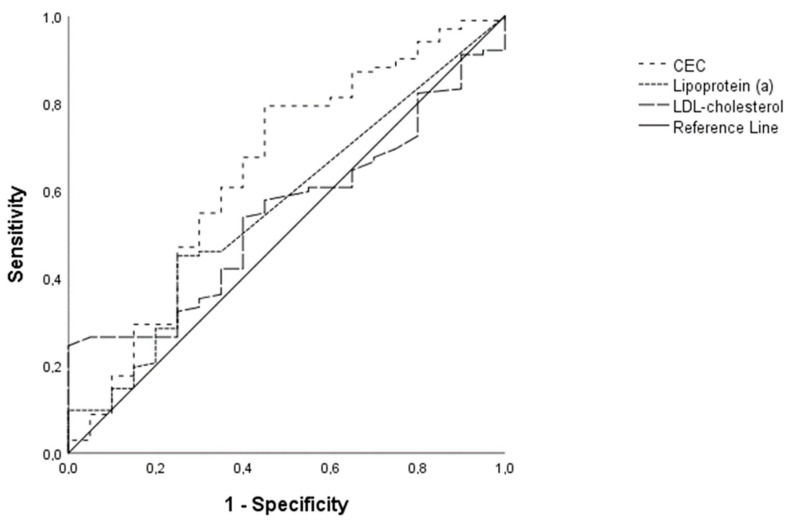
Receiver operating curves displaying the prediction of lower total plaque volume. The area under the curve (AUC) of CEC (0.65) was significantly higher than the AUC of low-density lipoprotein cholesterol (0.53) and lipoprotein (a) (0.56) in patients without lipid-lowering therapy. CEC = cholesterol efflux capacity, AUC = area under the curve.

Patients with low total PV showed significantly higher CEC values than patients with high total PV. The area under the curve of CEC for the prediction of high total PV (0.65, 95% CI 0.50–0.79; *p* = 0.039) was significantly higher compared to the one for LDL-C (0.55, 95% CI 0.43–0.67; *p* = 0.525) and lipoprotein (a) (0.56, 95% CI 0.43–0.70; *p* = 0.384) (Figure 3).

The optimal cut-off value of 89.11% revealed that CEC could predict a low total PV with 79.4% sensitivity and 55% specificity.

### 3.4. Multivariate Analysis

A multivariate regression model based on traditional cardiovascular risk factors, vascular diseases, and biomarkers was calculated to predict high total PV (Table 2).

Significantly univariate analysis parameters with *p*-values < 0.05 (age, sex, hypertension, hyperlipidemia, vascular disease, CKD, and CEC) were included in the multivariate analysis. The odds ratio for CEC was calculated for an increase of 1%, using CEC as a continuous variable.

The parameters of age and vascular diseases directly correlated with high total PV, whereas female sex showed a significant indirect correlation with high total PV. Unlike hypertension, hyperlipidemia, and chronic kidney disease, CEC remained borderline significant in the multivariate analysis.

## 4. Discussion

In this cross-sectional analysis of 176 subjects without current lipid-lowering therapy, we observed an inverse relationship between CEC and high total PV, which was evaluated by a 3D ultrasound in patients not taking lipid-lowering treatment. On the other hand, we did not discover any correlation between high total PV and lipoprotein (a), LDL-C, or CE transfer.

Our findings are in contrast to a subanalysis from the Multi-Ethnic Study of Atherosclerosis, which linked CEC to a greater risk of carotid plaque advancement [15]. In this work, CEC was measured in two cohorts with the respective age and sex-matched controls. Cohort 1 included patients with cardiovascular events during the 10-year follow-up period, while cohort 2 consisted of patients with plaque progression of the carotid artery measured after 10 years in two ultrasound examinations. Although a greater CEC level was linked to a faster advancement of carotid plaque in cohort 2, cohort 1 had a decreased risk of incident CVD, which was primarily driven by cardiac events linked to CAD. This study [15], in contrast to our investigation, used a scoring system rather than plaque volumetry to examine the evolution of plaques [15]. Inverse correlations of CEC with coronary events were also documented by other observational studies, including the Dallas Heart Study [11] and the European Prospective Investigation of Cancer (EPIC)-Norfolk study [14]. The Dallas Heart Study also revealed a negative correlation between CEC and incident strokes.

Other cross-sectional studies demonstrated an inverse relationship between CEC and carotid artery stenosis [35,36,37]. A similar inverse association between CEC and acute coronary syndrome was found in a recently published case-control study of 250 individuals. Low CEC was associated with a higher likelihood of developing acute coronary syndrome when antioxidative activity (reflected in paraoxonase 1 activity) was also low [38]. Contrary to these findings, a study following participants from a cardiac catheterization laboratory over 3 years showed a direct association of CEC and cardiovascular events [39]. In conflict with these data, our team demonstrated that CEC inversely correlates with cardiovascular mortality in patients with and without previous CVD, including those at high risk [17,18].

Experimental studies in mice provided initial evidence that reverse cholesterol transport may be inversely associated with arteriosclerosis [40]. Therefore, the capacity of HDL to transfer excess cholesterol from the periphery, such as the arterial wall, back to the liver was proposed as a new biomarker of HDL-C function and a surrogate for clinical outcomes. HDL-C has multiple atheroprotective functions. It is also critical for signaling in endothelial cells, where HDL can induce nitric oxide synthase and enhance endothelial repair, thereby initiating angiogenesis [41].

Our research evaluated the primary role of HDL in clearing free cholesterol from the plasma membrane of macrophages. To this end, we used a cholesterol efflux assay that was previously applied in several clinical studies [18,42]. According to the correlation between HDL-C and CEC, the test employed in this study mostly captured HDL-C-mediated CEC [1]. In the general population, low HDL-C levels constitute an independent risk marker for atherosclerotic CVD [3]. However, efforts to lower CVD by increasing HDL-C, e.g., by using CETP inhibitors, have not yet proved effective. Although there is a strong inverse association between HDL cholesterol and cardiovascular disease, the factors that control CEC are still poorly understood [43]. Therefore, we also assessed the transfer of CE from HDL to apoB-containing lipoproteins, mediated by CETP, which is a crucial step in the reverse cholesterol route. In our study participants, we did not see any correlation between total PV and CETP-mediated CE transfer.

CEC is the initial rate-limiting step in HDL-mediated reverse cholesterol transport, by which excess cholesterol is removed from peripheral cells. However, efforts to lower CVD by increasing HDL (e.g., using CETP inhibitors) have been unsuccessful so far.

A small study investigated the effect of niacin, which leads to a moderate increase in the ability of HDL to mediate CEC, and the effect of CETP inhibitors such as anacetrapib, which leads to a more significant increase in CEC [44]. However, a large meta-analysis with more than 117,000 patients found no effect by niacin, fibrate, or CETP inhibitors on all-cause mortality, the mortality rate from coronary heart disease, myocardial infarction, or stroke [5].

Our study had several limitations. First, the sample size of our study was relatively small, and the study was conducted at one single center. In addition, most of our patients were in the low-to-intermediate-risk population. Therefore, the findings of this study are probably not transferable to a high-risk population.

To conclude, our study shows an inverse relationship between high peripheral plaque volume and CEC, though not CE transfer, in patients not receiving lipid-lowering medication. This and other observations may help to increase our understanding of the individual steps of reverse cholesterol transport. However, the presented results should be confirmed and extended in future studies comprising larger patient collectives.

## Figures and Tables

**Table 1:** Features of the study population.

		Total Plaque Volume		
	Study Population *n* = 176	Low (*n* = 148, 0–500 mm^2^)	High (*n* = 28, 501–2048 mm^2^)	*p*
Age, years	64 (57–70)	62.5 (56–68)	71 (61.25–75)	**0.001**
Sex (female)	86 (48.9)	81 (54.7)	5 (17.9)	**<0.001**
Body mass index, kg/m^2^	24.80 (22.73–27.1)	24.65 (22.7–27.1)	25.28 (23.68–26.8)	n.s.
Hypertension, *n* (%)	92 (52.3)	71 (48)	21 (75)	**0.009**
Family history of CVD, *n* (%)	38 (21.6)	35 (23.6)	3 (10.7)	n.s.
Smoking (pack years)	9.51 (±15.82)	9.42 (±15.72)	9.96 (±16.61)	n.s.
Hyperlipidemia, *n* (%)	133 (75.6)	116 (78.4)	17 (60.7)	**0.047**
Diabetes mellitus, *n* (%)	15 (8.5)	13 (8.8)	2 (7.1)	n.s.
hs-CRP, mg/dL	0.18 (0.08–0.41)	0.17 (0.08–0.38)	0.21 (0.09–0.44)	n.s.
Total cholesterol, mg/dL	218 (191.5–247)	223 (194.5–250)	210 (178–230.25)	n.s.
LDL-C, mg/dL	139 (117–170)	143 (117.5–171.5)	132.5 (108.25–169.25)	n.s.
HDL-C, mg/dL	61 (48.5–76.5)	63 (51–77.5)	52 (43–65.5)	**0.009**
Triglyceride, mg/dL	123 (94.5–184.5)	122 (92.5–178)	128 (96.25–225.75)	n.s.
Lipoprotein (a), mg/dL	20 (20–51.25)	20 (20–56.95)	20 (20–36.48)	n.s.
Antihypertensive therapy, *n* (%)	72 (40.9)	56 (37.8)	16 (57.1)	n.s.
Antidiabetic therapy, *n* (%)	9 (5.1)	9 (6.1)	0 (0)	n.s.
CKD, *n* (%)	27 (15.3)	18 (12.2)	9 (32.1)	**0.007**
CVD, *n* (%)	31 (17.6)	20 (13.5)	11 (39.3)	**0.002**

Parameters are median (interquartile range) or mean (± standard deviation) as indicated for continuous variables or number (percentage) for categorical variables. CVD = cardiovascular disease, hs-CRP = high-sensitive C-reactive protein, LDL-C = low-density lipoprotein cholesterol, HDL-C = high-density lipoprotein cholesterol, CKD = chronic kidney disease, CVD = cardiovascular disease, n.s. = not significant. Statistical significant differences (*p* < 0.05) between Low and High Total Plaque Volume are shown in bold.

**Table 2:** The odds ratios for increased total plaque volume.

	High Total Plaque Volume	
**Multivariate Proportional Odds**	**OR (95% CI)**	***p* Value**
Age (years)	1.09 (1.02–1.16)	**0.014**
Sex (female)	0.12 (0.04–0.41)	**<0.001**
Hypertension	1.66 (0.54–5.13)	0.381
Hyperlipidemia	0.44 (0.15–1.26)	n.s.
Vascular disease	4.39 (1.40–13.72)	**0.011**
CKD	1.80 (0.52–6.21)	n.s.
CEC (%)	0.97 (0.95–1.00)	**0.040**

CKD = chronic kidney disease, CEC = cholesterol efflux capacity, OR = odds ratio. n.s. = not significant. Statistical significant (*p* < 0.05) odds ratios are shown in bold.

## Data Availability

The data are available on request.

## Supplementary Materials

The following supporting information can be downloaded at: https://www.mdpi.com/article/10.3390/biomedicines11071918/s1, Table S1: Features of the study population.

## Abbreviations

AUC	area under the curve
apo B	Apolipoprotein B
CAD	coronary artery disease
CE	cholesteryl esters
CEC	cholesterol efflux capacity
CETP	cholesterol ester transfer protein
CKD	chronic kidney disease
CVD	cardiovascular disease
eGFR	glomerular filtration rate
HDL-C	high-density lipoprotein cholesterol
LDL-C	low-density lipoprotein cholesterol
PV	plaque volume
