# Supplementary material for: Inverse Correlation of Cholesterol Efflux Capacity with Peripheral Plaque Volume Measured by 3D Ultrasound
Source: Biomedicines. 2023 Jul 6;11(7):1918. doi: 10.3390/biomedicines11071918 (PMC10376979; doi:10.3390/biomedicines11071918)
Supplement: Supplementary file 1 [file biomedicines-11-01918-s001.zip › biomedicines-2410306-supplementary.pdf]

Supplementary material

Table S1. Features of the study population

	**Original cohort study *n* = 442**	**Participants without lipid lowering therapy *n* = 176**	**Participants with lipid lowering therapy *n* = 266**	***p***
Age, years	64 (57-72)	64 (57-70)	64.5 (56-73)	n.s.
Sex (female)	184 (41.6)	86 (48.9)	98 (36.8)	**0.012**
Body mass index, kg/m2	25.6 (23.6 -28.4)	24.8 (22.73 -27.1)	26.2 (24.2 -28.86)	**<0.001**
Hypertension, *n*(%)	294 (66.5)	92 (52.3)	202 (75.9)	**<0.001**
Family history of CVD, *n*(%)	108 (24.4)	38 (21.6)	70 (26.3)	n.s.
Smoking (pack years)	12.89 (± 19.45)	9.51 (± 15.82)	15.26 (± 21.36)	**0.024**
Hyperlipidemia, *n* (%)	392 (88.7)	133 (75.6)	259 (97.4)	**<0.001**
Diabetes mellitus, *n*(%)	55 (12.4)	15 (8.5)	40 (15)	**0.042**
hs-CRP, mg/dl	0.18 (0.09-0.41)	0.18 (0.08-0.41)	0.19 (0.09-0.4)	n.s.
Total cholesterol, mg/dl	189 (160.75-223.25)	218 (191.5-247)	173 (149-197)	**<0.001**
LDL-C, mg/dl	113 (90-144)	139 (117-170)	98.5 (82-122)	**<0.001**
HDL-C, mg/dl	57 (46-71)	61 (48.5-76.5)	55 (45-66)	**<0.001**
Triglyceride, mg/dl	132 (95-179)	123 (94.5-184.5)	134 (97-177.5)	n.s.
Lipoprotein (a), mg/dl	20 (20-98.7)	8.33 (8.33- 21.36)	25.3 (20-132.6)	**0.018**
Antihypertensive therapy, *n*(%)	241 (54.5)	72 (40.9)	169 (63.5)	**<0.001**
Antidiabetic therapy, *n*(%)	44 (10)	9 (5.1)	35 (13.2)	**0.006**
CKD, *n*(%)	59 (13.3)	27 (15.3)	32 (12)	n.s.
CVD, *n*(%)	155 (35.1)	31 (17.6)	124 (46.6)	**<0.001**

Parameters are median (interquartile range) or mean (± standard deviation) as indicated for continuous variables or number (percentage) for categorical variables.

CVD = cardiovascular disease, hs-CRP = high-sensitive C-reactive protein, LDL-C = low density lipoprotein cholesterol, HDL-C = high-density lipoprotein cholesterol, CKD = chronic kidney disease, CVD = cardiovascular disease, n.s. = not significant. Statistical significant differences (p = 0.05) between participants with and without lipid lowering therapy are shown in bold.
